# Updating Experimental Models of Diabetic Cardiomyopathy

**DOI:** 10.1155/2015/656795

**Published:** 2015-04-20

**Authors:** J. Fuentes-Antrás, B. Picatoste, A. Gómez-Hernández, J. Egido, J. Tuñón, Ó. Lorenzo

**Affiliations:** ^1^IIS-Fundación Jiménez Díaz, Autónoma University, 28040 Madrid, Spain; ^2^Spanish Biomedical Research Centre in Diabetes and Associated Metabolic Disorders (CIBERDEM) Network, 28040 Madrid, Spain; ^3^Biochemistry and Molecular Biology Department, School of Pharmacy, Complutense University of Madrid, 28040 Madrid, Spain

## Abstract

Diabetic cardiomyopathy entails a serious cardiac dysfunction induced by alterations in structure and contractility of the myocardium. This pathology is initiated by changes in energy substrates and occurs in the absence of atherothrombosis, hypertension, or other cardiomyopathies. Inflammation, hypertrophy, fibrosis, steatosis, and apoptosis in the myocardium have been studied in numerous diabetic experimental models in animals, mostly rodents. Type I and type II diabetes were induced by genetic manipulation, pancreatic toxins, and fat and sweet diets, and animals recapitulate the main features of human diabetes and related cardiomyopathy. In this review we update and discuss the main experimental models of diabetic cardiomyopathy, analysing the associated metabolic, structural, and functional abnormalities, and including current tools for detection of these responses. Also, novel experimental models based on genetic modifications of specific related genes have been discussed. The study of specific pathways or factors responsible for cardiac failures may be useful to design new pharmacological strategies for diabetic patients.

## 1. Main Models of Diabetic Cardiomyopathy (DCM)

Experimental models of both type I and type II diabetes (T1DM and T2DM) consistently exhibit alterations in the circulating levels of glucose and in the lipid profile (Table 1). Main T1DM and T2DM animals show both hyperglycemia (early after pancreas-toxin or fat/sweet-diet administration, resp., and later after genetic mutations) and hyperlipidemia, represented by elevated levels of TAG, cholesterol, and lipoproteins. More interestingly, these models also exhibit functional, structural, and metabolic abnormalities that recapitulate the human DCM pathology.

*Dysfunction in Cardiac Mechanics*. In patients, early stages of DCM are marked by a deterioration of longitudinal systolic function, a compensative elevated radial function, and diastolic dysfunction [1]. DCM progression is characterized by reduced ejection fraction (EF) and ventricular dilatation and, later, mimicking dilative cardiomyopathy. Experimental T1DM and T2DM animals are prone to develop diastolic and/or systolic dysfunction, as demonstrated in numerous* in vivo* studies using echocardiography, magnetic resonance imaging (MRI), and hemodynamic measurements [2, 3]. Diastolic dysfunction usually precedes the alteration of cardiac contractility. As recently updated [4], a broad analysis of diastolic performance should include several Doppler indexes: ratio of peak velocity of early to late filling of mitral inflow (*E/A*) and deceleration time of early filling of mitral inflow, as well as the isovolumetric relaxation/contraction time. Given the subjectivity of visual echocardiographic estimations and the frequent coexistence of different Doppler severity patterns, measurement of left ventricular (LV) diastolic pressures by catheterization is also highly valuable. The main systolic parameters include LV septum thicknesses, LV internal dimensions, posterior wall (PW) thicknesses at diastole and systole, LV mass, LVEF, LV fractional shortening (FS), and ventricular contractility assessment (*dP/dt*). Regardless of rather inconstant particularities mainly displayed by Akita mice, all T1DM models have been reported to meet most of these criteria (Table 1). Cardiac performance has also been extensively studied in T2DM experimental models. Genetically obese mice displayed severe diastolic dysfunction, as evidenced by diminished E/A velocities in* ob/ob* and* db/db* mice [5, 6]. Contractile properties are yet marginally affected in* ob/ob* mice [5]. On the contrary,* db/db* mice exhibited a reduced FS and velocity of circumferential fibre shortening at age 12 weeks [6]. Van den Bergh et al. also reported in* db/db* a decreased preload recruitable stroke work, end-systolic elastance, and* dP/dt* from age 24 weeks onwards but found preserved cardiac output, EF, and* dP/dt* [7] (Table 1). Other T2DM models presented heterogeneous data regarding cardiac performance. In ZDF rats, impaired diastolic function has been extensively described [8–10]. However, Zhou's work initially reported a decrease of FS at age 20 weeks [8], and we and others found no evidence of systolic dysfunction by both echocardiographic and MRI determinations at ages 16 and 44 weeks, respectively [9, 10]. Similarly, OLETF and GK rats preserved contractile function while displaying abnormalities of ventricular relaxation, as suggested by a prolonged deceleration time, decreased peak velocity, and reduced LV diameters associated with increased LVPW thickness and LV mass [11, 12]. In contrast, diet-induced obesity appears to invariably associate both diastolic and systolic dysfunctions. High-fat diet increased LV mass and reduced* dP/dt* and FS after 6 weeks [13]. Alike, sucrose fed rats exhibited early abnormalities in LV fillings, as demonstrated by reduced E/A ratios, together with depressed FS and EF after 10 weeks [14]. Consistently, echocardiographic findings in other high-fat and high-sucrose fed mice revealed decreased FS, EF, and velocity of circumferential fibre shortening, as well as dramatically impaired parameters of diastolic function after 16 weeks [15].

Thus, cardiac dysfunction in T1DM patients could be roughly reproduced in all conventional models (induced by toxins and genetic alterations). In T2DM, diet-induced models may represent the human pathology more appropriately, at least in the advanced states of the disease.

*(1) Alterations of Cardiac Structure*. Maladaptive structural modifications underlie both the diastolic and systolic impairments that eventually lead to heart failure. Although the progression of these events has not been fully established, the hallmark of changes includes cell hypertrophy, local inflammation, and interstitial fibrosis promoted by steatosis and cell-death processes in the injured myocardium.

*(a) Hypertrophy*. Undiagnosed cardiac hypertrophy is prevalent in asymptomatic T2DM patients, reaching up to 56% in recent reports [16]. Myocyte hypertrophy is common in biopsy of diabetic hearts and is induced by long-standing metabolic imbalances and microcirculation anomalies. However, the contribution of cardiomyocyte hypertrophy to the development of ventricular hypertrophy is still unclear [17]. Most models of DCM display progressive concentric or eccentric hypertrophy, which is directly associated with diastolic and systolic derangement, respectively. Indicative parameters include LV mass and heart-to-body weight (or femur length) ratio, LVPW and septum thicknesses, LV internal diameters, and cardiomyocyte size. In addition, complementary plasma levels of brain natriuretic peptide (BNP), atrial natriuretic peptide (ANP), and *β*-myosin heavy chain (*β*-MHC) have traditionally been considered supportive [18]. However, a recent systematic analysis of hypertrophic biomarkers in diabetic rodents revealed that most commonly measured genes are confounded by diabetogenic methods and do not correlate with cardiac hypertrophy [19]. In STZ-induced models, reduced wall thicknesses and increased LV internal diameters are prominent features, accounting for extensive ventricular dilatational remodeling [20] (Table 1). We observed cardiomyocyte size enlargement in long-term T1DM (22 weeks), reproduced to a lesser extent in short-term counterparts (6 weeks) [21]. Similar histological findings have been reported in OVE26 mice together with the upregulation of ANP and *β*-MHC [22, 23]. Conversely, no evidence of myocardial hypertrophy has been found in Akita mice [24]. In T2DM mice models, though systolic dysfunction is developed as a late event, myocardial mass may increase earlier. A higher heart weight has been reported in* ob/ob* and* db/db* mice at ages 12 and 9 weeks, respectively [25, 26]. A comprehensive MRI analysis of* db/db* hearts by Yue et al. revealed a significant increase in LV mass and LVPW, and septum thicknesses, as soon as age 13 weeks [26]. However, early architectural changes are subtle and not consistently identified. In this line, echocardiographic and cell dimension assessments did not find hypertrophy in 15-week-old* db/db* mice, despite evidence of systolic compromise [27]. In contrast, ZDF and GK rats usually exhibited a significant increase of the septum and LVPW thicknesses, reduced LV diastolic and systolic diameters, enlarged cardiomyocyte volume, and higher expression rates of ANP, in spite of the fact that contractile function was frequently preserved [10, 12, 28–31]. Finally, the limited body of evidence on diet-induced T2DM rather pointed hypertrophy as the only morphological alteration constantly present, independently of its high-fat of high-sugar diet origin [13, 32–34]. However, a recent work reported no difference in heart weight and cardiomyocyte size but overexpression of ANP, BNP, and *β*-MHC after a 16-week-long high-fat diet [35].

*(b) Inflammation.* Chronic low-grade inflammation has been recently added to the features of DCM in human patients [36]. Inflammatory signaling in cardiomyocytes usually occurs as an early response to myocardial injury and entails an activation of the proinflammatory nuclear transcription factor-*κ*B (NF-*κ*B) and the related expression of cytokines (i.e., tumour necrosis factor-*α* (TNF*α*), interleukins (IL-1*β*, IL-6), and chemokines (i.e., MCP-1)), and adhesion molecules (i.e., intracellular and vascular cell adhesion molecule-1 (ICAM-1, VCAM-1, resp.)) [37, 38]. Toll-like receptors and inflammasome signalling platforms may also be key participants in DCM-associated inflammation [39]. In STZ-treated hearts, both macrophage and lymphocyte infiltration and increased expression of proinflammatory cytokines and adhesion molecules were detected at 22 weeks, but not 6 weeks, after injection [21] (Table 1). Likewise, Li's paper reported an augmented NF-*κ*B activity in OVE26 mice [22]. A comprehensive approach to myocardial inflammation in Akita mice was recently available, indicating the upregulation of TNF*α* and the attenuation of anti-inflammatory IL-10 [40]. However, data from T2DM models is more variable.* Ob/ob* and* db/db* mice displayed inflammation and increased myocardial proinflammatory factors [41], whereas we are not aware of any work addressing inflammation in the heart of GK and OLETF rats or KK A^y^ mice. ZDF myocardia showed either absence of inflammatory cell infiltrates at ages 9 and 13 months or higher levels of proinflammatory cytokines at age 22 weeks [42]. Inflammation is also present in diet-induced models of obesity and diabetes. Several studies on high-fat fed mice showed upregulation of proinflammatory factors, together with lower measurements of anti-inflammatory adiponectin and IFN*γ* [35, 43, 44]. In turn, high-sucrose fed mice did not present inflammation [32].

*(c) Fibrosis*. Interstitial fibrosis is the histologic hallmark of human DCM as a result of both replacement of focal myocyte death and response to inflammatory infiltrate. Accelerated extracellular matrix (ECM) deposition leads to ventricular stiffness in the diabetic heart, which also stimulates a local increase of cytokines and NF-*κ*B [45]. Collagen type I and type III fibres accumulate in the epicardial and perivascular domains, whereas type IV is mostly found in the endocardium. Collagen can further undergo glycation by advanced glycation end-products (AGEs) and impair its degradation, leading to fibrosis, myocardial stiffness, and decreased cardiac relaxation. Quantification of interstitial fibrosis may be evaluated by histological staining (Masson's trichrome, Azan, and Sirius red) and gene/protein expression of ECM components. Valuable insight into the molecular substrate of ECM deposition is provided by the analysis of TGF*β*-Smad-AP1 axis, poly(ADP-ribose) polymerase-1 (PARP-1), and matrix metalloproteinases (MMP) activity (MMP/TIMP (tissue inhibitor metalloproteinase) ratio). We reported that the myocardium of both short-term and long-term STZ-treated rats undergoes intense fibrotic remodeling, mainly at the expense of interstitial and perivascular ECM deposits and TGF*β* axis [21] (Table 1). OVE26 mice and BB rats also displayed myocardial fibrosis, as determined by increases in collagen accumulation and reduced MMP activity [22, 46]. As with hypertrophy, histological study of Akita mice showed no evidence of fibrosis and elevated levels of ECM proteins [24]. In T2DM, most models exhibited extensive myocardial collagen deposits. In ZDF rats, we and others described an enlarged interstitial space mostly confined to the subendocardial domain [10, 29]. Similarly, we recently reported increased ECM deposition and upregulated expression profibrotic factors in the GK myocardium [12]. Other models in which fibrosis has been clearly detected include* db/db* and* ob/ob* mice and OLETF rats [18, 26, 47]. On the contrary, several groups pointed the absence of increased collagen deposition and ventricular stiffness in* ob/ob* despite strong evidence of other DCM traits such as hypertrophy and apoptosis [5, 25]. Much less has been done so far to assess fibrosis in diet-induced obesity models, in which a complete histological examination is lacked. High-fat fed mice presented overexpression of TGF*β* axis and downregulation of antifibrotic p-Smad1/5 and bone morphogenetic protein-2 (BMP-2) [34, 43]. However, Nunes et al. found no differences in perivascular and interstitial fibrosis in high-sucrose fed mice compared to controls [32].

*(d) Steatosis*. Accumulation of fat in nonadipose tissues in human diabetes may be a protective response to provide a store of fuel for subsequent oxidation and to prevent exposure to toxic lipid metabolites such as ceramides [48]. However, a chronic imbalance of lipid storage versus lipid oxidation may lead to mechanical dysfunction. Insulin resistance and high levels of intramyocardial triacylglycerol (TAG), long-chain fatty acid (FA), TAG derivatives (i.e., diacylglycerols (DAG) and phospholipids), and ceramides are main triggers of heart disease in obesity and T2DM [49]. Also, ceramide and DAG can reduce insulin-stimulated signalling through the activation of PKC and NF-*κ*B. Surprisingly, myocardial steatosis is a common feature among T1DM models (Table 1). STZ-treated mice showed a 50% increase in cardiac content of FA [50], and both FA and ceramide deposits have been reported in NOD and Akita mice already at age 3 months [24, 51]. An association between increased myocardial lipid content and cardiac dysfunction has also been observed in rodent models of obesity and T2DM. Interestingly, Zhou's lab described that cardiac steatosis was evident in ZDF at 6 weeks even before the reduction of FS [8]. Also,* ob/ob* and* db/db* showed significantly elevated contents of phospholipids, whereas ceramides, cardiolipin, and unesterified cholesterol remained unaltered [5, 41, 52]. As expected, both high-fat and high-fructose-induced obesity models also displayed extensive myocardial steatosis [15, 53].

*(e) Apoptosis. *Myocardial cell death is recognized as a major event in the progression of DCM. Biopsies from diabetic patients revealed that cardiomyocytes are more susceptible to diabetes-induced apoptosis than endothelial cells or fibroblasts [54]. Standard methods to study apoptosis include TUNEL staining, phosphatidylserine exposure in cell membrane, and analysis of caspase-3 and other components of the extrinsic (i.e., Fas, FasL) and intrinsic (i.e., Bax/Bcl-2 ratio, cytochrome-C) apoptotic pathways. Of note, the DNA repairing enzyme PARP-1, which is also recognized as an apoptotic marker, has been recently shown to be central in a genuine type of cell death termed parthanatos [55], and its potential role in DCM has already been stated [56]. STZ-treated hearts displayed a significant increase in cardiomyocyte apoptosis already at 3 days after injection. A progressive decrease of apoptosis was reported from 4 to 24 weeks after the induction of diabetes [57] (Table 1). In this line, we showed that apoptosis is more severe in long-standing STZ-treated hearts than in short-term ones [21]. Similarly, apoptosis was stimulated in OVE26 [58]. T2DM hearts also augmented rates of cell death. Comprehensive studies on* ob/ob* and* db/db* mice reported marked pro-apoptotic molecules and apoptosis [59, 60]. Moreover, cell death became more accentuated with aging [46, 61–63]. Likewise, we and others have described spread apoptosis in ZDF and GK myocardia [10, 12, 64]. Finally, most of the works on high-fat and high-sugar fed mice reported increased myocardial apoptosis [13, 43, 61, 65]. However, no change in apoptosis markers was identified in a more recent contribution in high-sucrose fed mice [66].

In summary, inflammation in human DCM usually occurs in early stages of the disease. Main established T1DM and T2DM models could emulate this response when analysed soon after the onset of the pathology. Also, most of them (but Akita) can exhibit myocardial hypertrophy, fibrosis, steatosis and apoptosis, preferentially at late stages of the disease. Thus, these models may be used to reproduce specific human responses to DCM, in particular when obesity coexists in the patients.

**Figure 1:**
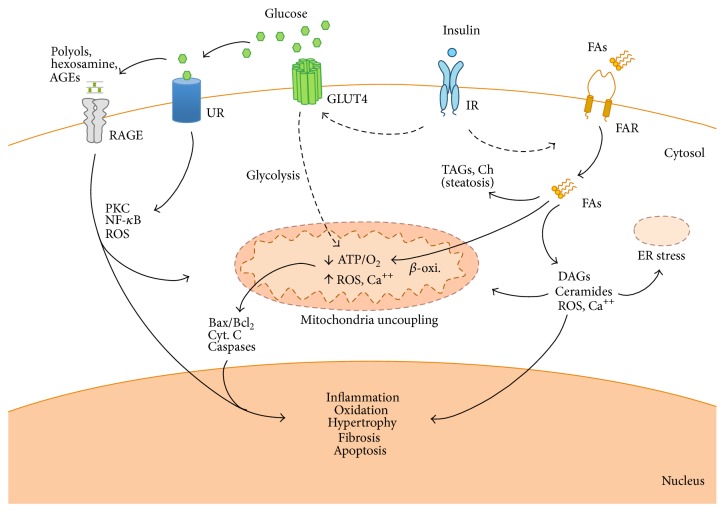
The diabetic milieu at the myocardium. Thickened lines represent activated pathways, and dotted lines denote reduced pathways. The absence of insulin response by defect in its secretion (T1DM) or sensibility (T2DM) promotes a lack of glucose assimilation for energy demand in the cardiomyocytes and extracellular glucose accumulation. Thus, FA may be the unique energetic substrate but *β*-oxidation may saturate and lipid can accumulate and cause steatosis and lipotoxicity by DAG, ceramide, and ROS formation. These metabolites together with glucose derivatives (i.e., polyols, AGEs) damage the mitochondria and endoplasmic reticulum (ER) and regulate the expression of proinflammatory, hypertrophy, fibrotic, and apoptotic genes. RAGE, receptor for advanced glycation end products; UR, unspecific receptors; IR, insulin receptor; FAR, fatty acid receptors. Ch: cholesterol; Cyt. C: cytochrome-C.

*(2) Alterations in Cardiac Metabolism and Calcium Handling*. The structural alterations in DCM may be originated by disruptions in energetic metabolism, which almost completely relies on FA and requires higher rates of O_2_ consumption. This scenario induces a progressive loss of cardiac efficiency by mitochondrial uncoupling and endoplasmic reticulum stress, and subsequent apoptosis and cardiac dysfunction [67] (Figure 1).

*(a) Energetic Imbalance.* In human DCM, FA constitute principally the unique energy source of fuel due to the insulin inefficiency and reduction of glucose transporters and glucose oxidation enzymes [68]. As a consequence, PPAR(*α*, *β*, and *γ*) transcription factors are stimulated to increase gene expression of those proteins involved in FA-uptake, -transport, and -oxidation (FAO) and ceramide production, in both peroxisomes and mitochondria [69]. Main models of T1DM [62, 70, 71] exhibited a reduced expression of GLUT1 and GLUT4, and higher levels of the glucose oxidation inhibitor pyruvate dehydrogenase kinase 4 (PDK4) (Table 1). Upregulation of PPAR*α* and FAO proteins has also been extensively described [62]. Likewise, Akita mice hearts showed lower glucose degradation and greater palmitate oxidation and expression of FA-transporters [24]. Nevertheless, most of the experimental data regarding metabolic abnormalities of the diabetic heart derive from T2DM models. Insulin resistance and higher FA utilization have been observed in* ob/ob* mice. As a result, myocardial O_2_ consumption rose, and cardiac performance substantially declined [64]. In the same model, a consistent decrease of key regulators of glucose metabolism such as phosphofructokinase (PFK) and GLUT-4 and increased expression of a set of PPAR*α* and PPAR*γ* independent genes involved in FA metabolism was reported [5]. In like fashion,* db/db* mice displayed a 40% decrease in glucose uptake, together with a considerable reduction in carbohydrate oxidation from age 10 weeks onwards. At this age, palmitate oxidation was shown to contribute over 90% to the cardiac FA production [26, 63]. In addition, we and others demonstrated the abnormal lipid metabolism of ZDF hearts, which include upregulation of genes involved in FA uptake, lipid reesterification, and *β*-oxidation [8, 10, 72]. Several studies ranging from gene expression assessment to quantitative positron emission tomography imaging have recently uncovered similar alterations in OLETF and GK rats [11, 31]. Diet-induced models have also disclosed similar disturbances. A recent work described an excessive FA metabolism in high-fat fed mice, supported also by upregulated PPAR*α* [15]. In the same model, Cole et al. found increased FAO rates [73], though Ussher's group observed no association between lipid accumulation and insulin sensitivity [74].

*(b) Mitochondrial Dysfunction and Oxidative Stress*. Energetic imbalance triggers defects of mitochondrial respiration and ROS overload in human diabetes. In both T1DM and T2DM, metabolic maladaptation contributes to ROS imbalance by affecting multiple enzymatic systems including NADH oxidase (NOX), endothelial NO synthase, and, mainly, the mitochondrial respiratory chain [68, 75]. In STZ-treated rats, mitochondrial damage was evidenced by loss of membrane potential, increases in ROS production, and reduction in antioxidant glutathione [76] (Table 1). Further proteomic analysis revealed marked alterations in the expression of 24 different cardiac proteins implicated in metabolic derangement and ROS excess, half of them located in the mitochondria [77]. Likewise, OVE26 mice showed altered mitochondrial function, reduced glutathione, and increased mitochondrial biogenesis [62, 78, 79]. A comprehensive study pointed that mitochondria of Akita mice also displayed respiratory defects, reduced cristae density, and increased volume at the mitochondria. However, despite morphological aberrations, mitochondrial number and antioxidant defences remained unchanged, and the absence of mitochondrial uncoupling was evidenced by unaffected ATP-to-O_2_ ratio [71]. In T2DM,* db/db* mice exhibited increased O_2_ consumption, mitochondrial ROS generation, and lipid peroxidation. Mitochondrial uncoupling was associated with upregulated FAO genes and electron transfer flavoproteins [80].* Ob/ob* mice and OLETF maintain unchanged levels of uncoupled proteins despite signal of mitochondrial dysfunction [81, 82]. Higher mitochondrial ROS production and lipid peroxidation rates have been also described in ZDF, OLETF, and GK rats [10, 83–85]. In addition, an elevation in antioxidant levels was demonstrated in ZDF rats [86]. Interestingly, ZDF rats neither displayed increased myocardial O_2_ consumption nor showed a significant reduction of mitochondrial biogenesis-related factors [10, 87]. Finally, mitochondrial uncoupling appears to be invariably present in cardiomyocytes from diet-induced obese mice. High-fat intake is associated with an increased burden of oxidized proteins, NOX and uncoupling-related factors, and antioxidant responses [43, 65, 73].

*(c) Altered Ca* ^*2+*^ *Mobilization*. A progressive dysregulation of Ca^2+^ handling underlies the relaxation-contraction defects in human DCM. Impaired Ca^2+^ signaling may be the consequence of altered sarcolemmal or sarcoplasmic Ca^2+^ pumps and channels and ryanodine receptors [88]. Cardiac mitochondria from STZ-treated rats decreased the capacity to stimulate ATP synthesis via stimulation of Ca^2+^ pumps, and, consequently, it abridged the relaxation during work load [89] (Table 1). Impaired contractility and reuptake of Ca^2+^ in cardiomyocytes from OVE26 mice were associated with reduced Ca^2+^ ATPase-60A (SERCA2a) levels and cytoplasmic Ca^2+^ clearance [90]. BB rats and Akita mice reported altered cardiac excitation-contraction coupling as well [24, 91]. In experimental T2DM models, the mechanisms explaining decreased Ca^2+^ uptake are not yet fully resolved. In* ob/ob*, Li et al. noted that SERCA2a was markedly modified by oxidation [92], whereas Van Den Bergh et al. showed increased SERCA2a levels with less affinity for Ca^2+^, which reduced contractile capacity [25]. In* db/db* mice, Belke's lab did not report significant variance in ryanodine receptor levels but lessened SERCA2a [93]. However, unchanged expression of SERCA2a has been described in ZDF [72] and intracellular Ca^2+^ transient was unaltered in GK cardiomyocytes [94]. In addition, cardiomyocytes from high-fat fed mice displayed a significantly elevated baseline intracellular Ca^2+^ and reduced Ca^2+^ decay rate, together with a marked decrease in SERCA2a expression [13, 65]. Further, sucrose fed rats showed reduced Ca^2+^ uptake [14], and fructose fed mice exhibited an attenuation of diastolic Ca^2+^ and Ca^2+^ transient amplitude, as well as SERCA2a expression [95].

Thus, the main T1DM and T2DM experimental models could replicate the metabolic alterations occurring in human hearts, including the energetic imbalance and mitochondrial and Ca^2+^ defects.

## 2. Cardiac Responses of DCM-Like Models Induced by Genetic Modification

Specific target genes have been modulated to create DCM-like models in rodents (Table 2). As detected in T1DM patients, cardiac dysfunction, hypertrophy, and fibrosis were demonstrated by upregulation of PKC*β* [96] or by downregulation of GLUT4 [97–99], phosphoinositide dependent kinase-1 (PDK1) [100], phosphoinositide-3 kinase (PI3K) [101], or glucokinase (GCK) [102] genes (Table 2). Apoptosis and inflammation were less common (only in PKC*β* and/or PI3K), and some metabolic controversies were described. For example, the lack of GLUT4 or the cardiac-specific insulin receptor (CIRKO) [103, 104] decreased both FA and glucose metabolisms, but it intriguingly elevated oxidative stress without reducing mitochondrial function. On the other hand, as observed in T2DM patients, a decline of cardiac function and increased myocardial hypertrophy, fibrosis, and steatosis were predominantly found in PPAR*α* [105, 106], PPAR*γ* [107, 108], long-chain acyl-CoA synthetase-1 (LCACS1) [109, 110], lipoprotein lipase (LPL) [111, 112], and fatty acid transport protein-1 (FATP1) [113, 114] overexpressed mice. Apoptosis was also presented in PPAR*γ*, LCACS1, and, perhaps, LPL null mice. Interestingly, all these responses were exhibited by adipose triglyceride lipase (ATGL) deletion [115, 116]. Also, by ablation of the brown adipose tissue in the transgenic model of uncoupling protein-diphtheria toxin A (UCPDTA) [117, 118], mice exhibited a T2DM-like phenotype though a potential rise of related cardiac steatosis and apoptosis was not assessed. However, metabolic changes in all these models were principally represented by an increase of FA oxidation. The glucose metabolism, mitochondrial function, and Ca^2+^ mobilization were unpredictably changed or were not analysed.

## 3. Major Animal Models Recapitulating the Human DCM Pathology

### 3.1. T1DM Associated DCM-Like Models

The STZ-administration could recapitulate the most human DCM features associated with T1DM (Table 1). The injection of this *β*-cell killer can induce cardiac dysfunction in parallel with typical myocardial alterations in the structure and metabolism. However, STZ may also injure nonpancreatic tissues such as brain, and the rapid and accentuated onset of T1DM (i.e., robust hyperglycemia, lipotoxicity, and cardiac apoptosis after only 2-3 days of injection) can diverge from the human nature. In this regard, an upregulation of calmodulin in OVE26 mice may develop a slower and more humanized T1DM-associated DCM, though the energetic imbalance should be further examined. However, this point mutation disagrees with the patient aetiology, in which an autoimmune response is usually the origin of the disease. In addition, the lack of insulin production in Akita leads to some uncommon responses in the myocardium such as the absence of hypertrophy and fibrosis and unchanged levels of oxidation in spite of mitochondrial dysfunction. Nonconventional T1DM models may also emulate the human disorder (Table 2). Aside CIRKO and GLUT4 metabolic controversies, the absence of other mediators for glucose assimilation as GCK may mirror the T1DM phenotype in patients. Also, the lack of PDK1 might reduce the activation of PI3K-dependent insulin signaling [119], leading to a useful T1DM-associated DCM model.

### 3.2. T2DM Associated DCM-Like Models

Traditional models of T2DM are those with an abolished leptin-system activity (Table 1). Considering leptin activities other than satiety control, these models develop obesity and an average onset of T2DM pathology, with increased hyperglycemia, insulin resistance, and hyperlipidemia, in parallel with cardiac dysfunction. They mostly show expected structural alterations as in the human disorder. However, a polygenic model could be more representative. In this regard, GK rats trigger similar responses but the lack of obesity and steatosis is not usually observed in clinic. More valid models of T2DM-associated DCM can be the DIO. In particular, high-fat diet emulates responses of the leptin-system defect, including obesity and steatosis, but not as a consequence of a single mutation. These effects can be additionally enhanced by coadministration of STZ [120], though tissue toxicity may be increased. Other nonconventional approaches may mimic human DCM in T2DM (Table 2). The upregulation of PPAR*α* or PPAR*γ* unexpectedly leads to similar actions to the lack of leptin stimulation. In spite of increased FA-transporters and FAO enzymes, the myocardium accumulates lipids and ROS. Likely, the level of other PPAR-regulated genes such as reesterification enzymes, glucose transporters, and proinflammatory cytokines could also explain those actions [121]. Moreover, ATGL, LPL, and FATP1 are involved in FA uptake and accumulation in the cardiomyocytes, and LCACS1 catalyses the first step of lipid biosynthesis. In this regard, an excess of FA and lipid content could increase lipotoxicity and subsequent cardiac failure, as occurs in T2DM patients.

## 4. Conclusions and Future Remarks

Since human trials focused on identifying the cardiovascular outcomes of tight glycemic control failed to show significant benefit [122], the prevention and treatment of DCM may not be limited to the restoration of systemic parameters. It should also include the preservation of cardiac structure and function along with the local metabolic balances (Figure 1). Thus, the knowledge and manipulation of specific key pathways and mediators in appropriate experimental models may be useful for studying new approaches for diabetic patients. Although the experimental milieu (genetic background, diets, and animal location) must be obviously considered, we may have valuable armamentarium to investigate key factors for DCM pathology. In relation with current data, the most appropriate T1DM-model induced by pancreas toxicity is achieved by STZ. However, unique (nontoxic) mutations on glucose-related genes (not on insulin) have demonstrated a DCM-like T1DM phenotype, as occurs with GCK. In addition, due to its possible origin as an autoimmune failure, T1DM could be ideally produced by triggers of specific autoimmune reactions or *β*-cell death (i.e., virus, modified genes). For T2DM-linked DCM, we may have more working options, including both T2DM-obesity and T2DM aetiologies. Since human T2DM is usually acquired with an unhealthy lifestyle and/or unknown polygenic mutations, high-fat fed models can be closer to that point mutations on leptin system or in lipid storage. Polygenetic mutations of obesity, such as KK A^y^, and OLETF, should be further characterized, and nonobese polygenetic mutated GK rats could facilitate the study of T2DM* per se*. However, we probably need diet-conditioned models of polygenetic mutations of T2DM to exemplify a* bona fide* approach to a related DCM.

## Figures and Tables

**Table 1:** Main experimental models of T1DM and T2DM and related plasma and cardiac characteristics.

	Type I diabetes						Type II diabetes							
	Toxics		Genetics				Genetics						DIO	
	STZ	Alloxan	OVE26	Akita	NOD	BB rat	ob/ob	db/db	ZDF	KK A^y^	OLETF	GK	Fat	Sweet
	(Antibiotic toxicity)	(Uric acid derivative toxicity)	(Calmodulin overexpression)	(*Ins*2^+/−^)	(Nonobese diabetic mouse)	(Nonobese diabetic rat)	(*Lep* ^−/−^)	(*Lepr* ^−/−^)	(*Lepr* ^−/−^)	(Polygenic obese + *A* ^*y*^)	(Polygenic obese)	(Polygenic nonobese)	(Diet-induced obesity and diabetes)	
**Serum profile**														
Hyperglycemia onset (d: days; w: weeks)	2 d after injection [123]	5 d after injection [123]	2-3 w [79]	5-6 w [71]	30 w [51]	12 w [124]	8–15 w [125]	4–8 w [125]	14 w [126]	16 w [127]	18 w [128]	3 w [129]	1 w [130]	1 w [14]
Hyperlipidemia	TG, Ch [123] LDL, HDL [131]	TG, Ch [123]	TG [79]	TG [71]	TG [132] Ch, HDL [133]	FFA [124]	TG, FFA [64] LDL, HDL [134]	TG, LDL, HDL [135]	TG, VLDL, LDL, HDL [136]	TG [137]	TG, Ch [11]	TG, FFA, LDL, HDL [12]	TG, FFA, Ch [138, 139]	TG, Ch [14]

**Cardiac abnormalities**														
*Functional *														
Diastolic function	↓[140]	↓[141]	↓[90]	↓[24]	↓[142]	↓[143]	↓[5]	↓[144]	↓[8–10]		↓[11]	↓[12]	↓[13, 144]	↓[14]
Systolic function	↓[140]	↓[141]	↓[90]	↓[145]/ ~[24, 71]	↓[146]	↓[147]	~[5]	↓[142]/ ~[148]	↓[8]/ ~[9, 10]		~[11]	~[12]	↓[13, 144]	↓[14]
*Structural *														
Hypertrophy	↑[20]		↑[22, 23]	~[24]		↑[46, 149]	↑[64]	↑[26]/ ~[27]	↑[10, 28]			↑[12, 30]	↑[13]	↑[32]
Inflammation	~[21]		↑[22]	↑[40]			↑[150]	↑[41]	↑[42]				↑[44]	~[32]
Fibrosis	↑[21]		↑[22, 23]	~[24]			~[25]	↑[41]/ ~[25]	↑[10, 29]	↑[151]	↑[11, 47]	↑[12]	↑[43]	~[32]
Steatosis	↑[21]			↑[24]	↑[51]		↑[5]	↑[5]	↑[8, 10]				↑[144]	↑[53]
Apoptosis	↑[21]		↑[58]				↑[52]	↑[59]	↑[10, 60]			↑[12]	↑[13, 65]	~[32]

**Metabolic alterations**														
Glucose oxidation	↓[70]			↓[24, 71]			↓[5, 64]	↓[26]	↓[10, 72]			↓[31, 80]	↓[74]	
FA oxidation	↑[70]		↑[62]	↑[24, 71]			↑[5, 64]	↑[63]	↑[10, 86]			↑[31]	~[74]/↑[73]	↑[53]
Mitoch. function	↓[70]		↓[62]	↓[71]			↓[81]	↓[152]	↓[10]	↓[151]	↓[83]	↓[84]	↓[73]	
Oxidative stress	↑[70, 76]	↑[153]	↑[78, 79]	~[71]			↑[92]	↑[152]	↑[10, 86]		↑[47, 83]	↑[84, 85]	↑[34]	↑[53]
Ca^2+^ mobilization	↓[70]		↓[90]	↓[24]		↓[91]	↓[25]	↓[93]	~[72]			↓[94]	↓[13, 95]	↓[14]

T1DM was induced by toxins [streptozotocin (STZ) or alloxan] or genetic mutations (OVE26, calmodulin transgenic; Akita, insulin-2 deficient; NOD, nonobese diabetic or BB, BioBreeding diabetes-prone mice). T2DM models were produced by genetic alterations [*ob/ob*, leptin deficient mice; *db/db*, leptin receptor deficient mice; ZDF, Zucker Diabetic Fatty rats; KK A^y^, yellow obese gene transgenic KK mice; OLEFT, Otsuka Long-Evans Tokushima fatty rats; GK, Goto-Kakizaki rats or DIO, diet-induced (fat, sweet) obesity].

**Table 2:** Genetic models of DCM and related cardiac responses.

	Upregulation							Downregulation					
	PPAR*α*	PPAR*γ*	LCACS1	LPL	FATP1	PKC*β*	UCPDTA	CIRKO	GLUT4	ATGL	PDK1	PI3K	GCK
Heart failure markers	↑ANP, BNP	↑ANP, BNP	↑ANP, BNP	↑ANP, BNP					↑ANP, BNP			↑ANP, BNP	

**Cardiac abnormalities**													
*Functional *													
Diastolic function	↓	↓	↓	↓	↓	↓	↓	↓	↓/~	↓	↓	↓	↓
Systolic function	↓	↓	↓	↓	~	↓	~	↓	↓/~	↓	↓	~	
*Structural *													
Hypertrophy	↑	↑	↑	↑	↑	↑	↑	↓	↑	↑	↑	↑	↑
Inflammation						↑				~			
Fibrosis	↑		↑	↑	↑	↑	↑	~	↑/~	↑	↑	↑	↑
Steatosis	↑	↑	↑	↑	↑			~	~	↑			
Apoptosis		↑	↑	↑/~		↑				↑	~	↑	

**Metabolic alterations**													
Glucose oxidation	↓	~		↓				↓	↓/~	↑	↓		↓
FA oxidation	↑	↑	↑	↑	↑		↑	↓	↓	↓	↑		↑
Mitochondrial function	↑	↑	↑				↑	↑		↓		↑	↑
Oxidative stress	↑	↑	↑					↑	↑			↑	↑
Ca^2+^ mobilization				~	~				↓		~		

References	[105, 106]	[107, 108]	[109, 110]	[111, 112]	[113, 114]	[96]	[117, 118]	[103, 104]	[97–99]	[115, 116]	[100]	[101]	[102]

Genetic overexpression of PPAR*α*, PPAR*γ*, long-chain acyl-CoA synthetase-1 (LCACS1), lipoprotein lipase (LPL), fatty acid transport protein-1 (FATP1), PKC*β* and uncoupling protein-diphtheria toxin A (UCPDTA), or ablation of cardiac-specific insulin receptor (CIRKO), GLUT4, adipose triglyceride lipase (ATGL), phosphoinositide dependent kinase-1 (PDK1), phosphoinositide-3 kinase (PI3K), and glucokinase (GCK) were forced in rodents for DCM. The associated cardiac effects and the levels of natriuretic peptides in plasma are also highlighted for each model. ↑, ↓ and ~ stand for increased, decreased, or not modified effect, respectively.

## Abbreviations

AGEs: Advanced glycation end products
ANP: Atrial natriuretic peptide
AP-1: Activating protein-1
ATGL: Adipose triglyceride lipase
Bax: Bcl-2-associated X protein
Bcl2: B-cell lymphoma 2
BMP-2: Bone morphogenetic protein-2
BNP: Brain natriuretic peptide
DCM: Diabetic cardiomyopathy
ECM: Extracellular matrix
EF: Ejection fraction
FA: Fatty acid
FAO: Fatty acid oxidation
FasL: Fas ligand
FATP1: Fatty acid transport protein-1
FS: Fractional shortening
GCK: Glucokinase
GLUT: Glucose transporter
ICAM-1: Intercellular adhesion molecule-1
IFN*γ*: Interferon-*γ*
IL: Interleukin
LCACS1: Long-chain acyl-CoA synthetase-1
LV: Left ventricular
MCP-1: Monocyte chemoattractant protein-1
MMP: Matrix metalloproteinase
MRI: Magnetic resonance imaging
NF*κ*B: Nuclear factor of *κ*-light polypeptide gene enhancer in B-cells
NOX: NADH oxidase
PARP-1: Poly(ADP-ribose) polymerase 1
PDK: Phosphoinositide dependent kinase
PI3K: Phosphatidylinositol 3-kinase
PKC: Protein kinase C
PPAR: Peroxisome proliferator-activated receptor
PW: Posterior wall
ROS: Reactive oxygen species
SERCA2a: Sarco(endo)plasmic reticulum calcium ATPase
TAG: Triacylglyceride
TGF*β*: Transforming growth factor *β*
TIMP: Tissue inhibitor of metalloprotease
TNF*α*: Tumor necrosis factor *α*
TUNEL: Terminal deoxynucleotidyl transferase- (TdT-) mediated dUTP nick end labelling
UCPDTA: Uncoupling protein- (UCP-) promoter-driven diphtheria toxin A
VCAM-1: Vascular cell adhesion molecule-1
*β*-MHC: *β*-Myosin heavy chain.
